# Aberrant O‐glycosylation contributes to tumorigenesis in human colorectal cancer

**DOI:** 10.1111/jcmm.13752

**Published:** 2018-07-12

**Authors:** Yuliang Jiang, Zhe Liu, Feng Xu, Xichen Dong, Yurong Cheng, Yizhang Hu, Tianbo Gao, Jian Liu, Lei Yang, Xingyuan Jia, Haili Qian, Tao Wen, Guangyu An

**Affiliations:** ^1^ Department of Oncology Beijing Chao‐Yang Hospital Capital Medical University Beijing China; ^2^ Medical research Center Beijing Chao‐Yang Hospital Capital Medical University Beijing China; ^3^ Key Laboratory of Molecular Oncology Cancer Institute and Hospital Chinese Academy of Medical Sciences Beijing China

**Keywords:** Colorectal cancer, MUC2, mucin, O‐glycosylation, Tn antigen

## Abstract

Aberrant O‐glycosylation is frequently observed in colorectal cancer (CRC) patients, but it is unclear if it contributes intrinsically to tumorigenesis. Here, we investigated the biological consequences of aberrant O‐glycosylation in CRC. We first detected the expression profile of Tn antigen in a serial of human CRC tissues and then explored the genetic and biosynthetic mechanisms. Moreover, we used a human CRC cell line (LS174T), which express Tn antigen, to assess whether aberrant O‐glycosylation can directly promote oncogenic properties. It showed that Tn antigen was detected in around 86% human primary and metastatic CRC tissues. Bio‐functional investigations showed that T‐synthase and Cosmc were both impaired in cancer tissues. A further analysis detected an occurrence of hypermethylation of Cosmc gene, which possibly caused its loss‐of‐function and a consequent inactive T‐synthase. Transfection of LS174T cells with WT Cosmc restored mature O‐glycosylation, which subsequently down‐regulated cancer cell proliferation, migration and apoptotic‐resistant ability. Significantly, the expression of MUC2, a heavily O‐glycosylated glycoprotein that plays an essential role in intestinal function, was uniformly reduced in human CRC tissues as well as in LS174T cells. These data suggest that aberrant O‐glycosylation contributes to the development of CRC through direct induction of oncogenic properties in cancer cells.

## 1 INTRODUCTION

Colorectal cancer (CRC) is one of the most common human malignancies and the third leading cause of cancer‐related death worldwide.^1^, ^2^, ^3^ So far, the pathogenesis of CRC is less well understood. Somatic mutations in key proto‐oncogenes or tumour suppressors such as *Ras*,* APC* and *P53* have been proposed to play a tumour‐initiating role.^4^ In addition, many studies have shown that aberrant O‐glycosylation of proteins and lipids, resulting in exposure of the immature truncated O‐glycans such as Tn antigen,^5^, ^6^ is associated with tumorigenesis and malignant transformation in many human cancers including CRC,^7^ pancreatic cancer,^8^ breast cancer,^9^ liver cancer^10^ and ovarian cancer.^11^

Mucin‐type O‐glycosylation, which is the most common post‐translational modification of many membrane bound and secreted glycoproteins, occurs in the Golgi apparatus and is regulated by a series of glycosyltransferases.^12^ Normally, the biosynthesis of O‐glycans is initiated by polypeptide α‐N‐acetylgalactosaminyltransferases (ppGalNAc‐Ts), a family of enzymes which add GalNAc to Ser/Thr residues to form the Tn antigen.^13^ Tn antigen is the only precursor for all mucin‐type O‐glycans. For further elongation, T‐synthase (core 1 β1,3‐galactosyltransferase) is the key enzyme during the O‐glycosylation process.^14^ Interestingly, the expression and activity of T‐synthase require an endoplasmic reticulum(ER)‐resident molecular chaperone named Cosmc.^15^, ^16^, ^17^ Knockout of either T‐synthase or Cosmc in mice causes Tn expression in vivo.^18^, ^19^, ^20^ Besides, in gastrointestinal tract, Tn antigen can also be elongated into core 3 O‐glycans by specific glycosyltransferase‐C3GnT.^21^ Overall, T‐synthase, Cosmc and C3GnT are essential for complete O‐glycosylation in colonic tissues. Accordingly, aberrant O‐glycosylation in colonic tissues, characterized by Tn antigen exposure, has been suggested to arise from disturbances in the expression and activity levels of T‐synthase, Cosmc and/or C3GnT.^17^, ^22^, ^23^, ^24^, ^25^ However, it is not known whether or how aberrant O‐glycosylation may contribute to the development and progression of CRC. Here, we first reported that aberrant expression of truncated O‐glycans in the form of Tn antigen expression was seen in over 85% of human colorectal cancers. We then performed exome sequencing of the key genes responsible for O‐glycosylation and found no mutations in O‐glycosylation genes. Notably, we observed a high frequency of the hypermethylation of the Cosmc gene in CRC tissues, which may provide an explanation for the detected aberrant O‐glycosylation. We next asked whether aberrant O‐glycosylation caused by Cosmc dysfunction plays a causal role for the development of CRC. We used a human CRC cell line (LS174T), which harbours mutations in Cosmc and has a resultant inactive T‐synthase leading to Tn expression, to demonstrate that aberrant O‐glycosylation appears to directly enhance oncogenic features including altered cell growth, migration and apoptotic‐resistant properties in cells.

## 2 MATERIAL AND METHODS

### 2.1 Clinical specimens and cell line

Human primary/metastatic colorectal cancer and adjacent normal tissues were acquired from patients at Beijing Chao‐Yang Hospital, Capital Medical University, Beijing, China. The study was approved by the Ethics Committees of Beijing Chao‐Yang Hospital, Capital Medical University in accordance with the Declaration of Helsinki. Each patient provided a written consent. Human colorectal cancer LS174T cell line (Tn‐positive) was kindly provided by Dr. Tongzhong Ju of Emory University School of Medicine in Atlanta, Georgia, USA.

### 2.2 Immunohistochemical staining of Tn antigen

The formalin‐fixed, paraffin‐embedded tissues (n = 186) were cut into 5 μm sections. The sections were firstly stained with haematoxylin and eosin (H&E) using a standard protocol. For immunohistochemistry of Tn antigen, deparaffinized sections were boiled for 20 minutes in 0.01 mol/L citrate buffer at pH 6.0 for epitope retrieval. The sections were blocked using 0.3% H_2_O_2_ and 5% BSA. Then, sections were incubated overnight at 4°C with a specific anti‐Tn IgM monoclonal antibody (5 μg/mL, CA3638, clone 12A8‐C7‐F5, kindly provided by Dr. Tongzhong Ju, Emory University, Atlanta, Georgia, USA)^13^, ^20^, ^26^ followed by horseradish peroxidase‐conjugated antimouse IgM antibody (Abcam, ab97230) for 1 hour at room temperature. Finally, the sections were developed with DAB reagent (ZSGB‐BIO, China) and counterstained with haematoxylin.

### 2.3 Exome sequencing

Genomic DNA was isolated by DNA Isolation Kit (QIAGENInc.Valencia, CA, USA) and fragmented into 180‐280 bp by sonication (Covaris). The exon enrichment library was constructed under the manufacturer's instructions of Agilent Sure Select XT Kit (Agilent, CA, USA). The exome sequencing of 99 genes was performed by Illumina HiSeq2500 platform. Data were aligned to human genome UCSC hg19 by BWA and recalibration was performed by Picard and GATK. The Unified Genotyper module was used to detect SNP/INDEL.

### 2.4 Analysis of DNA methylation by MALDI‐TOF mass spectrometry

The isolated genomic DNA was bisulphate converted using EZ‐96 DNA methylation kit (Zymo Research, Orange, CA, USA). CpG islands of Cosmc and T‐synthase were identified through NCBI Gene Browser (http://www.ncbi.nlm.nih.gov/gene). DNA methylation primers were designed by EpiDesigner software (http://epidesigner.com). The following methylation‐specific primers were used: Cosmc‐Forward 5′‐ATTTTTATGTTAGGAAGGTGAAATGG‐3′, Cosmc‐Reverse 5′‐TCCTAACCAAACTATTCTAACTACAAAC‐3′, T‐synthase‐F 5′‐GGTGATTTTTGTTTTTTTGGGTAGT‐3′, T‐synthase‐R 5′‐TCAAAATCTTAAAACTAATACATAACCTT‐3′. An additional T7 promoter tag was added to each reverse primer for in vivo transcription, and a 10‐mer tag was added to the forward primer to balance the PCR primer length. PCR products were treated with Shrimp alkaline phosphatase (SAP) to dephosphorylate the unincorporated dNTPs. At the same time, the RNaseA was added to cleave the transcripts (T‐cleavage). The DNA methylation was quantitatively analysed by the MassARRAY platform (SEQUENOM).

### 2.5 T‐synthase activity assay

The assessment of the T‐synthase activity was conducted using a fluorescence‐based assay.^27^ T‐synthase from the tissue lysates will form non‐fluorescent Galβ1‐3GalNAc‐α‐(4‐methylumbelliferone (4‐MU)) by transferring Gal from UDP‐Gal to GalNAc‐α‐(4‐MU). Galβ1‐3GalNAc‐α‐(4‐MU) can be cleaved by an O‐glycosidase to release free 4‐MU. The fluorescence intensity of 4‐MU at high pH represents the activity of T‐synthase. In brief, tissues extracts were prepared using extraction buffer (25 mmol/L Tris‐HCl (pH 7.4) containing 150 mmol/L NaCl and 0.5% Triton‐X100). Then, a total volume of 40 μL master mixes containing 500 μmol/L GalNAc‐α‐4‐(MU), 500 μmol/L UDP‐Gal, 20 mM MnCl_2_, 0.1% Triton X‐100, 800 units of O‐glycosidase, 50 mmol/L MES‐NaOH buffer (pH 6.8) was placed in a 96‐well black plate. Add 10 μL of tissue extracts to each well and incubated at 37°C for 1 hour. Then, 100 μL of 1.0 mol/L Glycine‐NaOH (pH 10.0) was added to stop the reaction and the fluorescence intensity was assayed by a fluorimeter (Ex 255 nm/Em 460 nm). The fluorescence intensity of 4‐MU represents the amount of T‐synthase products as well as the T‐synthase activity. Reaction mix without UDP‐Gal was used as a control.^27^

### 2.6 RNA extraction and quantitative real‐time PCR

Total RNA was extracted from frozen CRC tissues and cells using TRIzol reagent (Invitrogen, CA, USA), according to the manufacturer's instructions. The RNA quality was assessed by a NanoDrop 2000 spectrophotometer (Wilmington, USA). Cosmc and T‐synthase mRNA levels were measured by qPCR using SYBR Premix (Applied Biosystems) on the 7500 Sequence Detection System (Applied Biosystems). GAPDH was used as an internal control. The sequences of primers were as follows: COSMC forward, 5′‐ACTGCAGCCCAAAGACTCACATCT‐3′, COSMC reverse, 5′‐ATGCACCACCATGAGCATCATCAC3′; T‐synthase forward, 5′‐TCTTACAGAAATACACTTTCGG‐3′, T‐synthase reverse, 5′‐ATTTTAACACACTTCACAGCTC‐3′; GAPDH forward, 5′‐AATCCCATCACCATCTTCCA‐3′,GAPDH reverse, 5′‐TGGACTCCACGACGTACTCA‐3′. Relative changes in mRNA were normalized with GAPDH and calculated using 2^−▵▵CT^ methods.

### 2.7 Western blotting

Tissues or cells were lysed with RIPA lysis buffer. Total protein concentrations were measured with BCA assay kit (Thermo Fischer). Samples containing equal amount of denatured protein were separated by 10% SDS‐PAGE, transferred onto PVDF membranes (Millipore) and probed with primary antibodies and horseradish peroxidase‐conjugated secondary antibodies. The detection was performed by ECL kit (Millipore) according to the manufacturer's instruction. The following primary antibodies were used: Cosmc antibody (2 μg/mL, Santa Cruz, sc‐271829), C3GnT antibody (1 μg/mL, Sigma, SAB1307144), C1GALT1 (T‐synthase) antibody (2 μg/mL, Santa Cruz, sc‐100745), β‐actin (Cell Signaling Technology, 8457) or GAPDH (1 μg/mL, Cell Signaling Technology, 5174).

### 2.8 Lentiviral‐mediated Cosmc transfection

The GV367‐EGFP‐Cosmc vector encoding Cosmc and the control vector GV367‐EGFP‐control were purchased from Shanghai Genechem Co. LTD (China). The Cosmc expression vector and the control were transfected into LS174T cells with polybrene (Genechem, Shanghai, China). The transfected cells were cultured for 2 days and then selected with puromycin.

### 2.9 Flow cytometry analysis

The cells (1 × 10^5^) were resuspended in 100 μL PBS and then incubated with anti‐Tn IgM mAb (20 μg/mL, CA3638, clone 12A8‐C7‐F5) at 4°C for 1 hour followed by incubation with PE‐labelled goat antimouse IgM (Santa Cruz, sc‐3768) for an hour. After washing twice with PBS, the cells were analysed by flow cytometer (BD bioscience). Similarly, for analysis of MUC2 expression, the cells were incubated with anti‐MUC2 antibody (10 μg/mL, Santa Cruz, sc‐15334) at 4°C for 1 hour followed by incubation with PE‐labelled secondary antibody (Santa Cruz, sc‐3739) and further analysed by flow cytometer (BD bioscience).

### 2.10 Cell proliferation, migration and apoptosis

The cell proliferation was measured by the Cell Count Kit‐8 (CCK‐8). The 1 × 10^4^ cells were seeded into 96‐well plates and incubated at 37°C. Before assay, medium was removed, 10 μL of the CCK8‐solution was added to each well of the plate and incubated at 37°C for 2 hours. The optical densities (OD) were determined at 450 nm by spectrophotometer.

Cell migration was determined using wound healing assay and transwell assay. Briefly, 2 × 10^6^ cells were seeded into a 6‐well plate. After the cells confluence reached approximately 90%‐100%, streaks were created with a pipette tip. The wells were then washed, and serum‐free medium was added. The cells were observed and photographed at 24 and 48 hours after wounding. For migration assays, serum‐free medium containing 2 × 10^5^ cells were added into the upper chamber (BD Bioscience, 8 μm pore size). After 24 hours, the cells migrated through the pore were fixed in 4% paraformaldehyde and stained with 0.1% crystal violet. The cells were counted and imaged under a microscope.

Apoptotic cells were examined using the PE Annexin V Apoptosis Detection Kit I (BD Biosciences). In brief, appropriate amount of cells were seeded into 35 mm tissue culture dish (Corning, 430165) with 2 mL complete medium. When the confluence reached around 80%‐90%, the cells were treated with UV irradiation for 10 seconds.^28^, ^29^ After 24 hours’ incubation, cells were collected and added with 5 μL of PE Annexin V and 5 μL of 7‐AAD and incubated for 15 min at room temperature in the dark. Finally, 400 μL 1 ×  Binding Buffer was added to each tube and the occurrence of apoptotic cells was measured by flow cytometer (BD Biosciences).^30^

### 2.11 Multiplex IHC staining

Totally, 48 formalin‐fixed paraffin‐embedded sections of human CRC tissues were stained with Tn antigen and mucin MUC2 simultaneously performed with Opal^TM^ 4‐Color Manual IHC Kit (PerkinElmer). Briefly, the sections were first incubated with a specific anti‐Tn IgM mAb(CA3638, clone 12A8‐C7‐F5) for 1 hour at room temperature and then incubated with Goat antimouse IgM mu chain (HRP)(Abcam, ab97230) and eventually labelled with Opal 520 fluorophore (PerkinElmer). For the staining of MUC2, Goat anti‐MUC2 antibody (2 μg/mL, Santa Cruz, sc‐3739) was applied and Opal polymer HRP Ms+Rb (PerkinElmer) was subsequently added to the sections followed by incubation with Opal 690 fluorophore (PerkinElmer). Finally, all sections were counterstained with DAPI and mounted with anti‐fade mountants. The inForm software (PerkinElmer) was used for analysing the multispectral images.

### 2.12 Statistical analysis

All data were analysed with the SPSS 19.0 statistical software (SPSS, Chicago, IL, USA) and GraphPad Prism 6.0 (GraphPad software, La Jolla, CA, USA). All real‐time PCR reactions were repeated three times. The relative quantity of the gene expression was normalized to GAPDH using 2^−▵▵ct^ methods. The methylation level was analysed by EpiTYPE software(SEQUENOM). Continuous data were presented as the mean ± SEM and analysed with Student's *t*‐test. Categorical data were analysed by chi‐square test. *P *<* *.05 were considered statistically significant.

## 3 RESULTS

### 3.1 Aberrant O‐glycosylation is frequently detected in human primary and metastatic CRC

**Figure 1:**
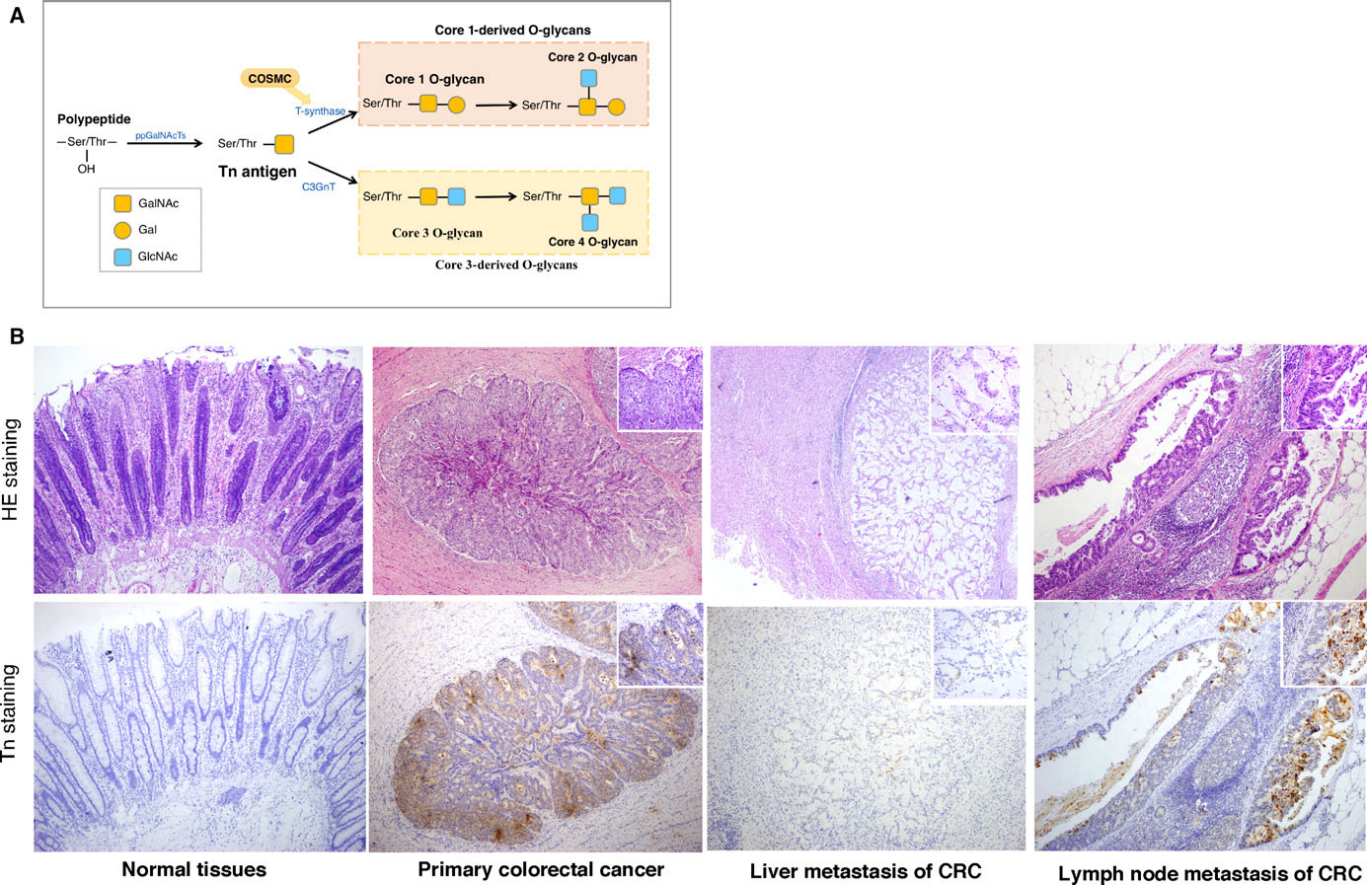
Expression of aberrant O‐glycans in human colorectal cancer. A, Biosynthesis of Tn antigen, and Core 1‐ and Core3‐derived O‐glycans. Tn antigen is either modified by T‐synthase and its chaperone (Cosmc) to form Core 1‐derived O‐glycans or processed by C3GnT to form the Core 3 structure. B, Representative H&E staining and immunohistochemical staining of Tn antigen in human primary and metastatic colorectal cancer tissues. Brown indicates positive staining for Tn antigen

To investigate the clinical relevance of aberrant O‐glycosylation, we first checked the expression of Tn antigen in archived paraffin‐embedded human CRC tissues. We observed that colonic samples in 161 of the 186 (86.6%) human CRC patients were Tn positive, where all non‐tumour tissues were Tn negative (Figure 1). Tn antigen was most often stained in the apical cell membranes, mucin droplet and the cytoplasm of the cancer tissues (Figure S1), suggesting a frequent occurrence of aberrant O‐glycosylation. Furthermore, we analysed whether there was an association between Tn antigen expression and the clinicopathological characteristics of CRC. The results showed that Tn antigen was significantly correlated to moderate histological differentiation (*P *=* *.022) and venous invasion (*P *<* *.001) of the tumours, while it was not related to age, gender, tumour location, invasive depth, status of lymph nodes, perineural invasion and TNM stage (Table 1). Although a further large‐scale systematic patient study is required, these promising data support that aberrant O‐glycosylation occurs predominantly in a subset of CRC patients, and may serve as an aetiological factor for the development of CRC.

**Table 1:** Correlation of Tn antigen with clinicopathological features in patients with colorectal cancer

Variable	Number of Patients	Tn expression		χ^2^	*P* value
		n	%		
Age					
<60	68	58	85.3	0.147	.701
≥60	118	103	87.2		
Gender					
Male	120	106	88.3	0.915	.339
Female	66	55	83.3		
Tumour location					
Colon	90	82	91.1	3.11	.078
Rectum	96	79	82.2		
Histological type					
Well differentiated	57	46	80.7	7.60	.022^a^
Moderate differentiated	92	86	93.4		
Poor differentiated+mucinous	37	29	78.4		
Invasive depth					
Mucosa/submucosa	46	38	82.6	0.90	.638
Muscle layer	54	48	88.9		
Subserosa/serosa exposed	86	75	87.2		
Lymph nodes metastasis					
Absent	98	82	83.6	1.48	.223
Present	88	79	89.8		
Venous invasion					
Absent	58	42	72.4	14.50	<.001^a^
Present	128	119	92.9		
Perineural invasion					
Absent	36	29	80.6	1.38	.24
Present	150	132	88.0		
TNM Stage					
I+II	88	79	89.8	1.48	.223
III+IV	98	82	83.7		

The chi‐square test was used to analyse the associations between factors.

a *P *<* *.05 was considered to be statistically significant.

### 3.2 Exploration of the mechanisms leading to Tn antigen expression in human CRC

**Figure 2:**
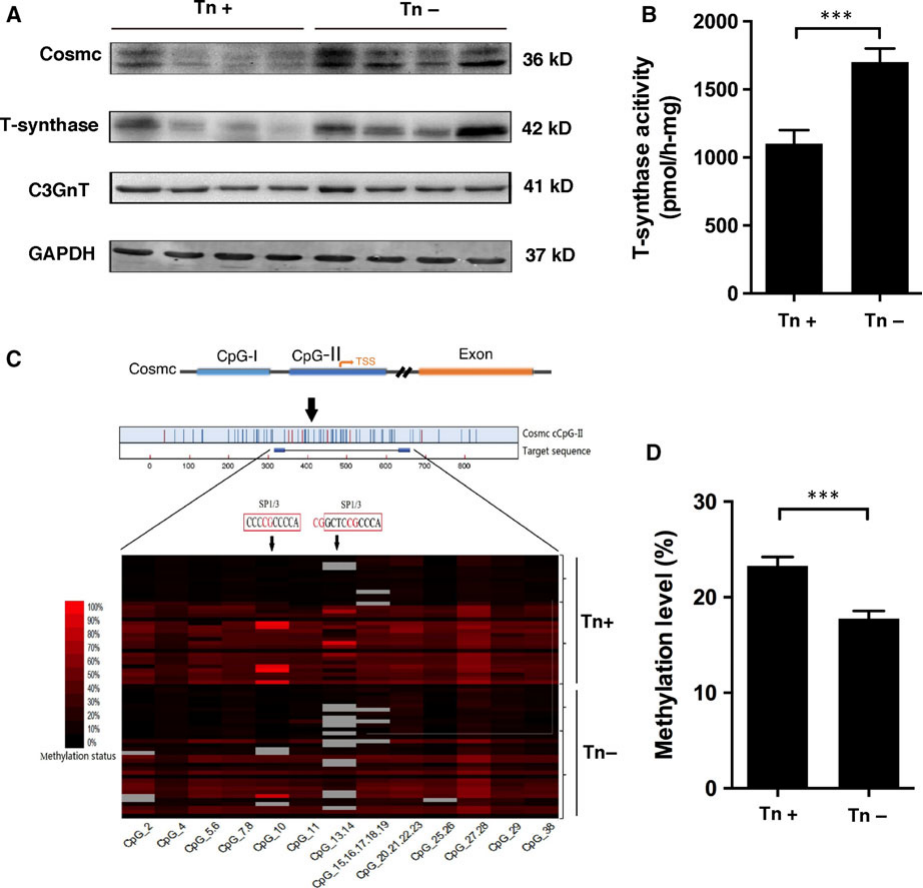
T‐synthase and its chaperone (Cosmc) are impaired in human colorectal cancer tissues expressing Tn antigen. A, Expression of T‐synthase, Cosmc and C3GnT in frozen human colorectal cancer tissues (Tn‐positive = 29, Tn‐negative = 22) was measured by western blotting. B, T‐synthase enzymatic activity was measured in human colorectal cancer tissues (Tn‐positive = 32, Tn‐negative = 26). C, Analysis of Cosmc DNA methylation by MALDI‐TOF mass spectrometry (Tn‐positive = 33, Tn‐negative = 32). D, Methylation levels of Cosmc in Tn‐positive and Tn‐negative cancerous tissues. ***P <* *.001

We further investigated the mechanisms responsible for Tn antigen expression detected in patients with CRC. It is known that mature O‐glycosylation depends specifically on T‐synthase activity and its single chaperone‐Cosmc.^31^ Besides, in colonic tissues, defective core 3 O‐glycosylation could also lead to the exposure of Tn antigen.^21^, ^32^ Here we found that the expression of C3GnT that controls core 3 O‐glycosylation in cancer tissues expressing Tn antigen (Tn‐positive) was comparable to that in Tn‐negative cancerous tissues, thereby suggesting that there were only possible defects in T‐synthase and/or Cosmc (Figure 2A). As expected, we showed that the expression and activity of T‐synthase were much lower in Tn‐positive CRC tissues relative to Tn‐negative cancerous tissues (Figure 2A,B). Concomitantly, the expression of Cosmc, the key chaperone required for T‐synthase activity and expression, was also found to be decreased in these Tn‐positive cancer tissues (Figure 2A). In addition, ppGalNAc‐Ts catalyze the initial O‐glycosylation step,^13^ so the changes in the ppGalNAc‐Ts expression may affect Tn expression. Here, we detected no significant differences in the mRNA levels of a range of ppGalNAc‐Ts between Tn‐positive and Tn‐negative tissues (Figure S2), thus ruling out a possible involvement of ppGalNAc‐Ts. Taken together, these data indicated that aberrant O‐glycosylation detected in CRC tissues may result from defects in T‐synthase and Cosmc.

It has been reported that somatic mutations and/or deletions in T‐synthase and Cosmc may be responsible for exposure of Tn antigen.^23^, ^33^, ^34^ We therefore performed exome sequencing to detect if there were genetic alterations in T‐synthase or Cosmc. Notably, an exome‐sequence analysis of Tn‐positive cancerous tissues exhibited no mutations in T‐synthase, Cosmc or C3GnT, all of which are essential for mature O‐glycosylation in colonic tissues (Figure S3). By contrast, a high frequency of somatic mutations was detected in *TP‐53*,* APC*, and *K‐ras*, etc., which was in agreement with published literature.^4^ These findings suggest that genetic alterations in T‐synthase, Cosmc and/or C3GnT are not prevailing mechanisms for such a high incidence of aberrant O‐glycosylation in CRC tissues.

Recently, hypermethylation in Cosmc, which would result in Cosmc gene silencing, was reported in pancreatic tumours and Tn syndrome expressing truncated O‐glycans.^26^, ^35^ We therefore conducted a MALDI‐TOF spectrometry methylation analysis of Cosmc and T‐synthase in another pair of Tn‐positive and Tn‐negative cancer tissues. We found that hypermethylation was predominantly present in Cosmc gene in Tn‐positive cancer tissues (n = 33) in contrast to Tn‐negative cancer tissues (n = 32; Figure 2C,D). No hypermethylation of T‐synthase gene was detected (data not shown). Our results suggested that hypermethylation of Cosmc gene may result in its loss‐of‐function, which consequently causes inactive T‐synthase and impairs O‐glycosylation process in CRC.

### 3.3 Aberrant O‐glycosylation enhances oncogenic properties in CRC cells

**Figure 3:**
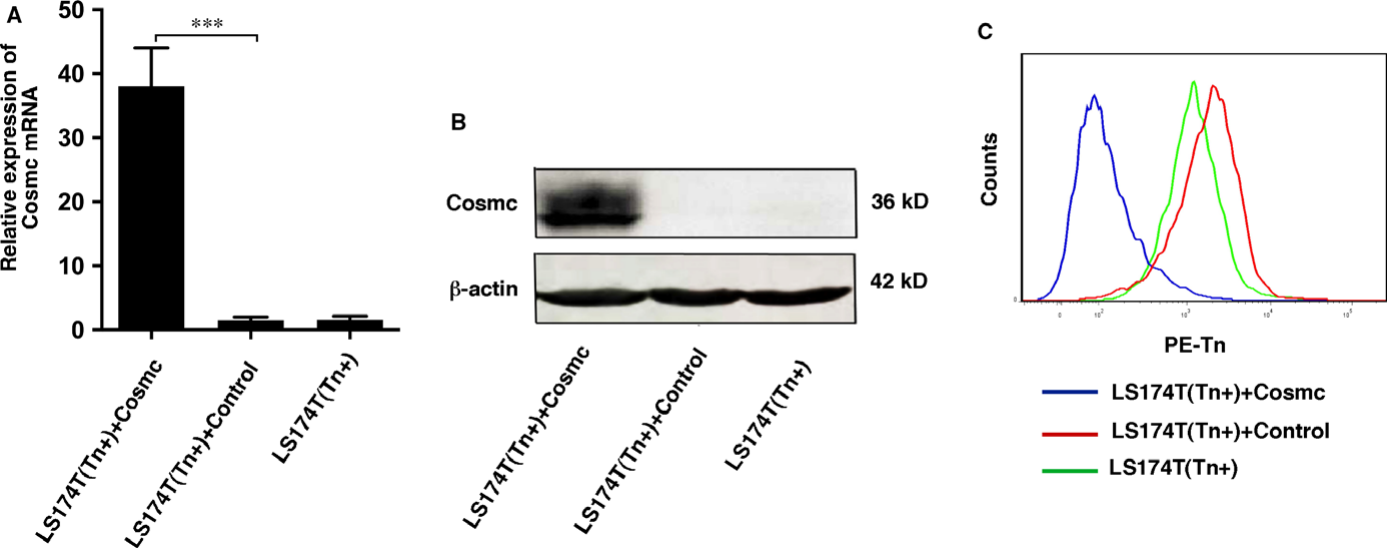
Transfection of WT Cosmc in LS174T cells. A, qPCR analysis of Cosmc mRNA levels in LS174T cells transfected with WT Cosmc or blank vector. B, Western blot analysis of Cosmc expression in LS174T cells transfected with WT Cosmc or blank vector. C, FACS analysis of Tn antigen in LS174T cells transfected with WT Cosmc or blank vector. All experiments were repeated for 5 or 6 times. ***P <* *.001

**Figure 4:**
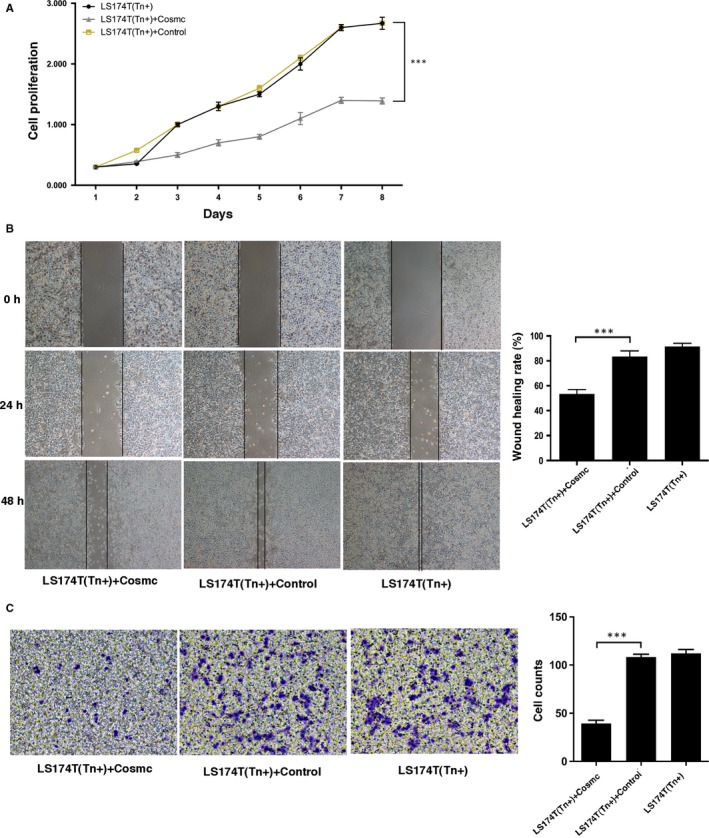
Cosmc transfection inhibits cell proliferation and migration in LS174T cells. A, Comparison of proliferation in LS174T cells transfected with WT Cosmc or blank vector. B, Wound healing assay in LS174T cells transfected with WT Cosmc or blank vector was performed to measure cell migration. Representative photographs (left) and quantification (right) were shown. C, Transwell migration assay was performed in LS174T cells transfected with WT Cosmc or blank vector. Representative photographs (left) and quantification (right) were shown. All experiments were repeated for 5 or 6 times. ***P <* *.001

**Figure 5:**
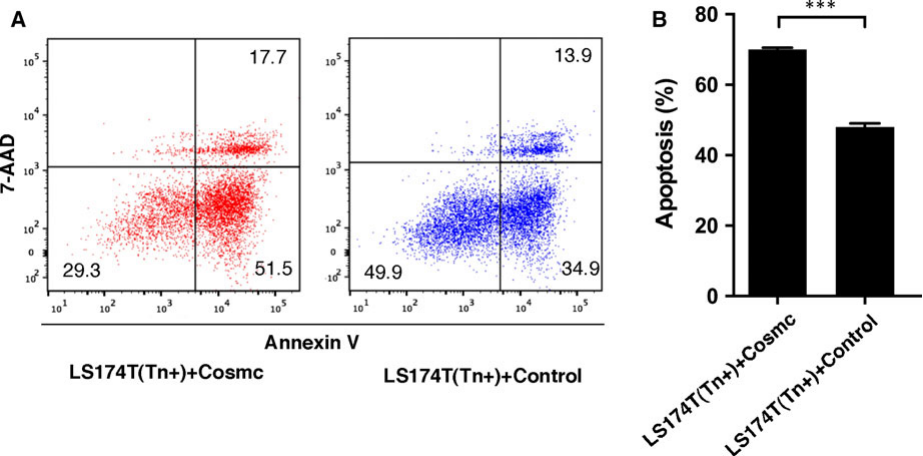
Cosmc transfection promotes UV‐induced apoptosis in LS17T cells. A, Representative images of FACS analysis of cell apoptosis in LS174T cells transfected with WT Cosmc or blank vector. B, Quantification of apoptosis rate in LS174T cells transfected with WT Cosmc or blank vector following UV treatment. All experiments were repeated for 5 or 6 times. ***P <* *.001

Human colorectal carcinoma LS174T cells harbour mutations in Cosmc gene and have expression of truncated O‐glycans such as Tn antigen.^23^ We used LS174T cells to evaluate the biological consequence of aberrant O‐glycosylation. LS174T cells were stably transfected with a plasmid encoding WT Cosmc gene. WT Cosmc transfection restored T‐synthase activity and abrogated the Tn antigen expression in these cells, indicating that the WT Cosmc could correct mature O‐glycosylation (Figure 3). We then explored the biological behaviours of LS174T cells after restoration of mature O‐glycosylation. The results showed that LS174T cells transfected with WT Cosmc (Tn‐negative) exhibited a prominent suppression in growth and migration properties as compared with blank vector‐transfected cells that still express Tn antigen (Tn‐positive) (Figure 4), suggesting that lack of proper O‐glycosylation enhances cancer cell proliferation and migration. In addition, we evaluated whether aberrant O‐glycosylation has an influence on cell apoptosis. We treated LS174T cells with ultraviolet (UV) light to induce a significant occurrence of cell apoptosis. We found that transfection of WT Cosmc remarkably increased the rate of cell apoptosis as compared to blank vector‐transfected cells (Figure 5), thus suggesting that aberrant O‐glycosylation endows cancer cells with significant apoptotic‐resistant ability, which may favour tumour development.

### 3.4 Aberrant O‐glycosylation correlates with impairment of MUC2 expression

We further investigated how aberrant O‐glycosylation affects cancer cell behaviours. It is known that mucins, primarily MUC2, are the predominant O‐glycoproteins in the colon.^36^ MUC2 is a major component of the intestinal mucus layer and heavily modified by O‐glycosylation. Altered expression of MUC2 has long been documented in patients with colorectal cancer and correlated with metastasis and prognosis.^37^, ^38^, ^39^, ^40^ We therefore hypothesize that aberrant O‐glycosylation may contribute to tumorigenesis and metastasis of cancer cells by affecting MUC2 expression and function.

**Figure 6:**
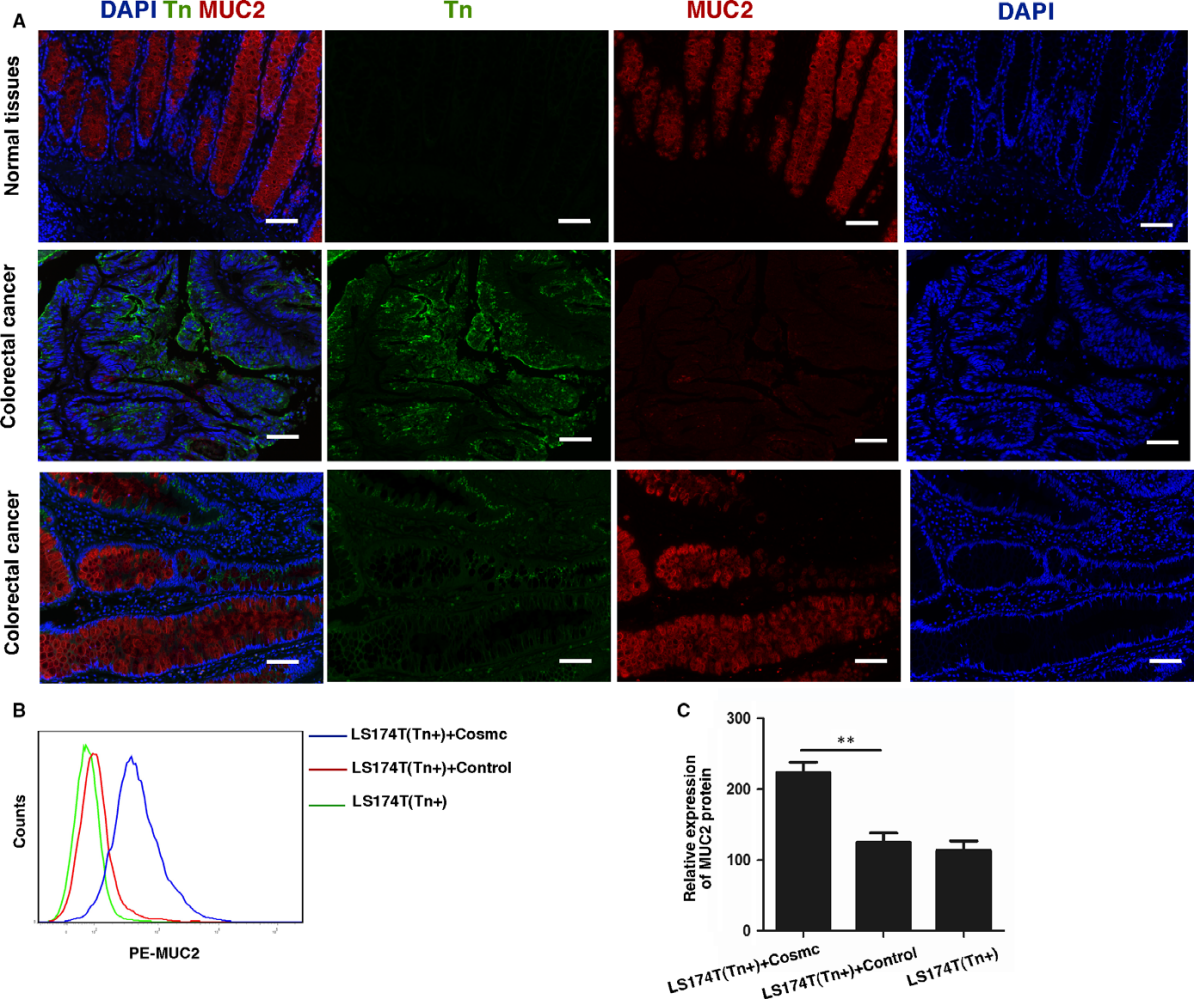
Tn antigen expression impairs MUC2 expression in human colorectal cancer tissues. A, Tn antigen and MUC2 expression were detected simultaneously in human CRC tissues (n = 48) by multiple immunohistochemical staining. There was an inverse correlation between Tn antigen and MUC2 expression. Scale bar, 100 μm. B, FACS analysis of MUC2 protein expression in LS174T cells transfected with WT Cosmc or blank vector. C, Quantification of MUC2 protein expression in LS174T cells by FACS. **P <* *.01

In human CRC tissues, we observed that the expression of Tn antigen was inversely correlated with MUC2 expression (Figure 6A), suggesting that aberrant O‐glycosylation may impair MUC2 expression. However, the decreased levels of MUC2 expression could arise from destructed epithelia as a result of severe inflammation/ulceration.^41^, ^42^ It is uncertain whether MUC2 deficiency is a primary response to Tn antigen expression or secondary to inflammation. Therefore, we used LS174T cells to address whether aberrant O‐glycosylation plays an aetiological role in the impairment of MUC2. A FACS analysis showed that LS174T cells containing aberrant O‐glycosylation have less MUC2 expression than LS174T cells with normal O‐glycosylation (Figure 6B,C), which is consistent with our clinical observation. Interestingly, we found that the mRNA levels of MUC2 were markedly increased in Tn‐positive LS174T cells compared with Tn‐negative cells (Figure S4), which may be due to a negative feedback regulation. These data demonstrate that mature O‐glycosylation is required for the expression and/or function of its essential glycoprotein MUC2.

## 4 DISCUSSION

Expression of aberrant forms of O‐glycans such as Tn antigen has been frequently observed in patients with CRC^7^, ^43^ and reported to be associated with tumour metastatic potential and poor prognosis.^44^, ^45^ However, whether defective O‐glycosylation occurs as a result of tumorigenesis or plays a contributing role in tumour development is unclear. In this study, we found that Tn antigen was frequently (over 85%) expressed in a series of clinical specimens of colorectal cancers, suggesting an involvement of aberrant O‐glycosylation in CRC. More importantly, we found that hypermethylation rather than somatic mutations in Cosmc was a major cause for expression of the Tn antigen. Cosmc is required for accurate folding of T‐synthase in ER.^15^ Our results were comparable with a recent report that Cosmc hypermethylation was a prevalent mechanism for aberrant expression of immature truncated O‐glycans in pancreatic cancers.^35^ However, other mechanisms such as Golgi to ER relocation of the enzymes initiating O‐glycosylation^46^, ^47^ and altered expression of enzymes responsible for sialylation cannot be excluded and may play distinctive roles under different circumstances,^48^ which await further exploration.

So far the key question that remains unsolved is whether the expression of truncated O‐glycans in cancer intrinsically has biological functions for the malignant process. The major challenge is how to find the real counterparts, for example Tn‐positive versus Tn‐negative tumour cells. It has been reported that human colorectal carcinoma cell line (LS174T) is a mixture of Tn‐positive and Tn‐negative cells.^23^ Studies have shown that Tn‐positive LS174T cells have inactive T‐synthase activity, which are caused by alterations in Cosmc, and transfection of WT Cosmc can restore T‐synthase activity and repair O‐glycosylation. Therefore, this cell model is most ideal to study how the O‐glycosylation in tumour cells alters their behaviours. Our results clearly showed that Tn‐positive cancer cells have significantly enhanced growth and invasion ability in contrast to Tn‐negative cells. Moreover, we demonstrated that Tn‐positive cells are more resistant to UV‐induced apoptosis than Tn‐negative cells. Our findings provided direct evidence that aberrant O‐glycosylation plays a contributory role in the induction of oncogenic features in colorectal cancer. However, it should be noted that there are some contradictory reports regarding the biological role of aberrant O‐glycosylation in a few malignancies. For example, Gao et al reported that loss of intestinal O‐glycans significantly promotes spontaneous duodenal tumours in mice and aberrant O‐glycosylation may serve as critical determinants of duodenal cancer risks^49^; however, Bergstrom et al unexpectedly found that, although mice deficiency in intestinal O‐glycans developed typical colonic cancers, it was indeed dependent on colitis rather than aberrant O‐glycosylation.^50^ In addition, several studies showed that direct deletion of Cosmc in pancreatic cancer cell lines induces classical oncogenic features, which is also in agreement with our findings.^35^, ^51^ But Huang et al reported that forced overexpression of Cosmc in human colorectal cancer cell lines significantly increases malignant behaviours, whereas knockdown of Cosmc suppresses oncogenic features.^52^ Although it is unclear how these discrepancies occur, it has been unanimously accepted that altered O‐glycosylation is closely associated with malignant phenotypes. The functional role of aberrant O‐glycosylation in CRC remains to be further elucidated. Our study adds a new dimension to this theme by demonstrating that Cosmc dysfunction in colonic cancer is sufficient to induce aberrant O‐glycosylation and consequently promotes oncogenic features, thereby supporting that aberrant O‐glycosylation contributes to the progression of CRC.

Having demonstrated that aberrant O‐glycosylation is a contributing factor in the development and progression of CRC, we next asked how aberrant O‐glycosylation alters cancer cell behaviours. Theoretically, aberrant O‐glycosylation may produce global effects on post‐translational modification of many proteins and lipids, and subsequently alters their functions involved with oncogenic properties.^13^ Of these O‐glycosylated proteins, the secretory mucin MUC2 is stored in bulky apical granules of the goblet cells and is a most important factor maintaining intestinal homeostasis.^38^ As the predominant O‐glycoprotein, MUC2 is heavily modified by O‐glycosylation and is generally considered to be essential for epithelial protection. MUC2‐lacking mice spontaneously developed colitis and colorectal cancer.^53^ Therefore, we assume that defective O‐glycosylation may affect the expression and/or function of MUC2 that is required for suppressing intestinal cancer. Our results showed that MUC2 expression was much reduced in Tn‐positive human colonic cancer tissues relative to normal tissues, suggesting that MUC2 may require mature O‐glycosylation for its proper expression and stability. However, the alterations in MUC2 may also be due to the loss of epithelia as a result of severe inflammation/ulceration. To specifically address this issue, we compared the production of MUC2 in LS174T cells and found that the expression of MUC2 was substantially reduced in Tn‐positive cells than in Tn‐negative cells, which supports our clinical observation that aberrant O‐glycosylation directly impairs MUC2 expression. Nevertheless, it should be noted that defective O‐glycosylation may cause more profound effects than dysfunction of an individual O‐glycoprotein such as MUC2. More efforts are needed to decipher which key pathways or glycoproteins are most often affected by truncation of O‐glycans in oncogenesis.

In summary, we provide evidence for a causal role of aberrant O‐glycosylation in the development of CRC, which is probably caused by hypermethylation of Cosmc, the key chaperone for T‐synthase activity. Aberrant O‐glycosylation may alter oncogenic behaviours in colorectal carcinomas by impairing the expression or stability of intestinal mucins, primarily MUC2. The molecular mechanisms deserved further investigation.

## CONFLICT OF INTEREST

The authors declare that they have no conflict of interest.
