# Supplementary figures and images for: Aberrant O‐glycosylation contributes to tumorigenesis in human colorectal cancer
Source: J Cell Mol Med. 2018 Jul 12;22(10):4875–85. doi: 10.1111/jcmm.13752 (PMC6156240; doi:10.1111/jcmm.13752)

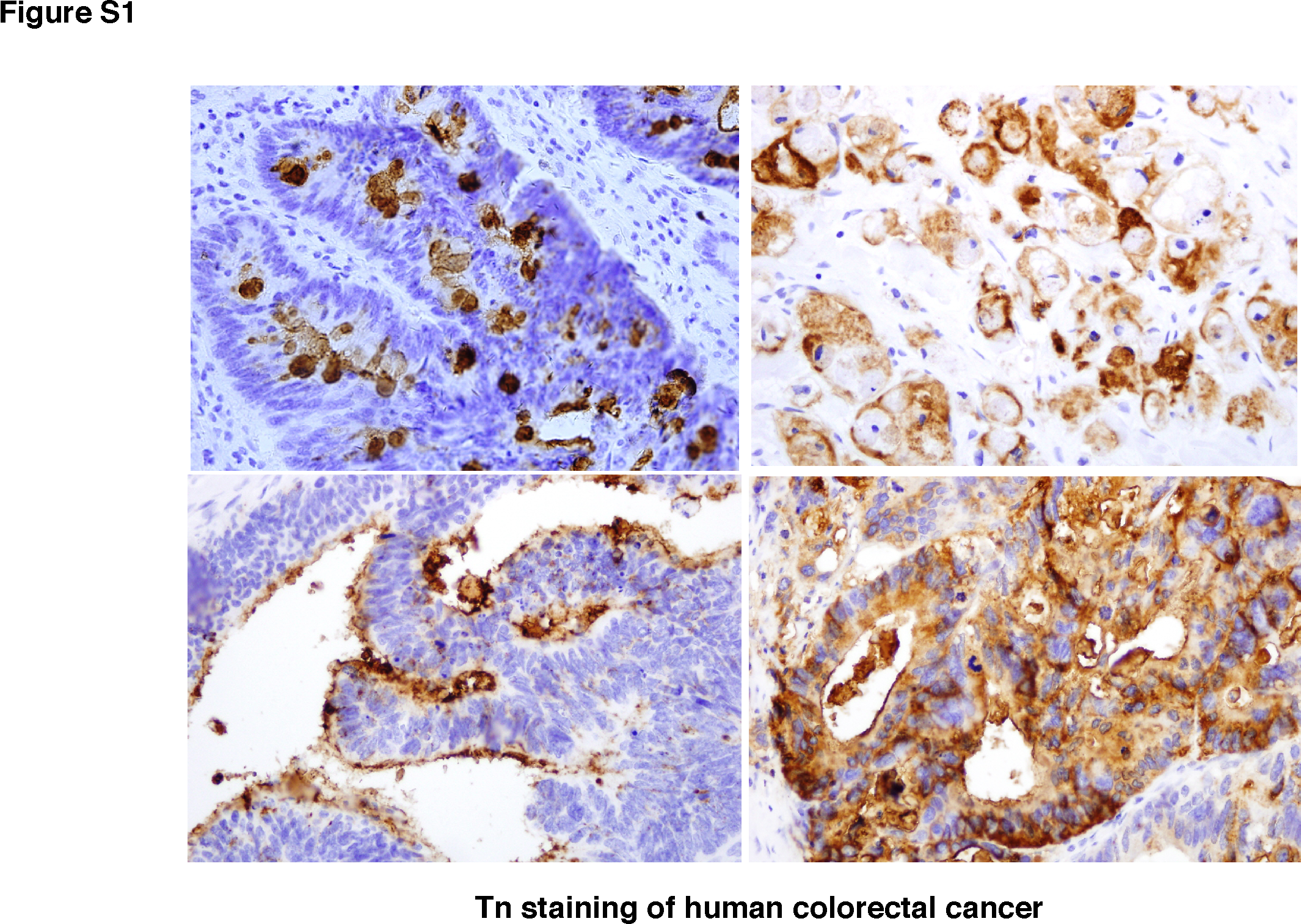

Supplement: Supplementary file 1 [file JCMM-22-4875-s001.tif]

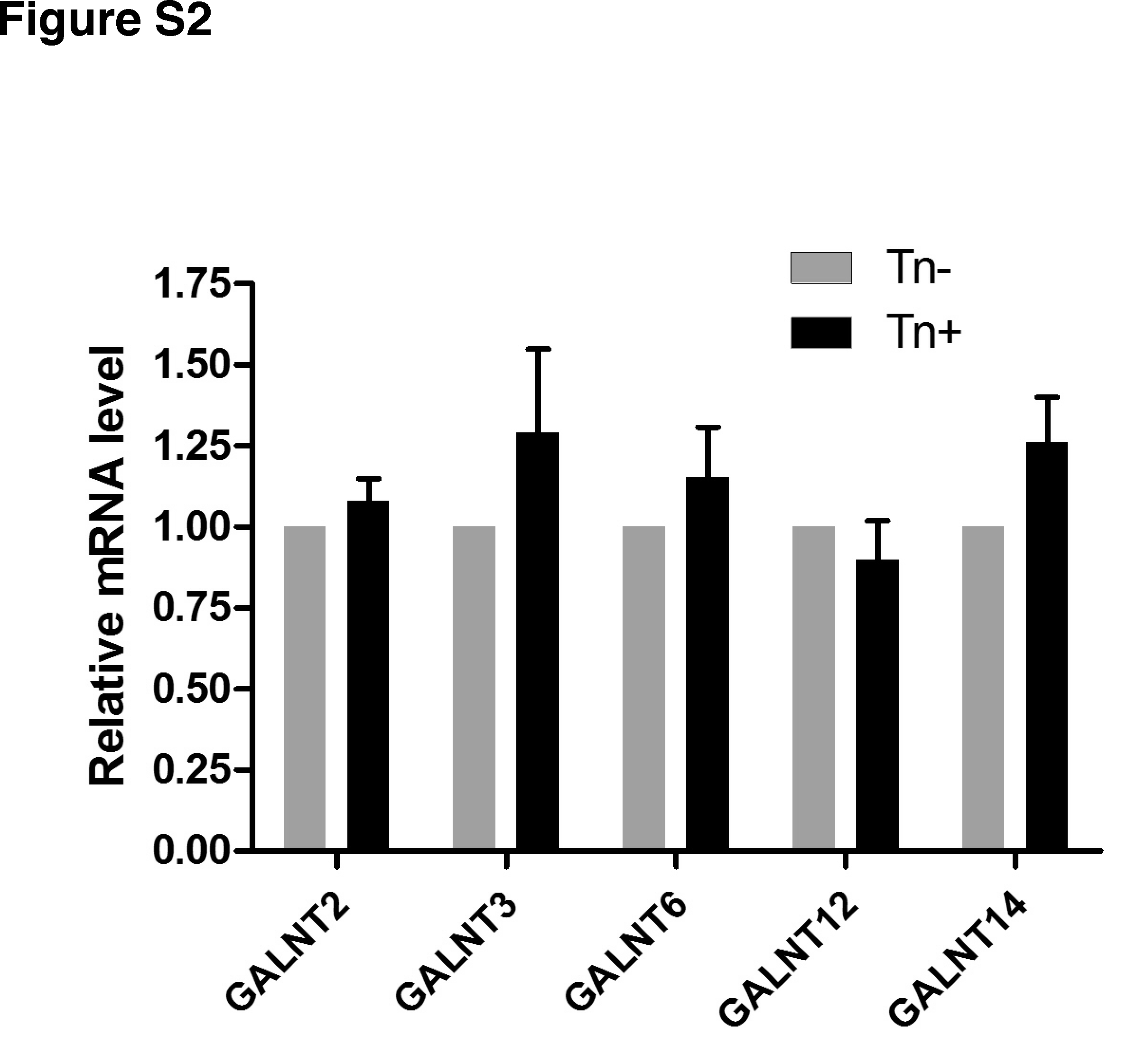

Supplement: Supplementary file 2 [file JCMM-22-4875-s002.tif]

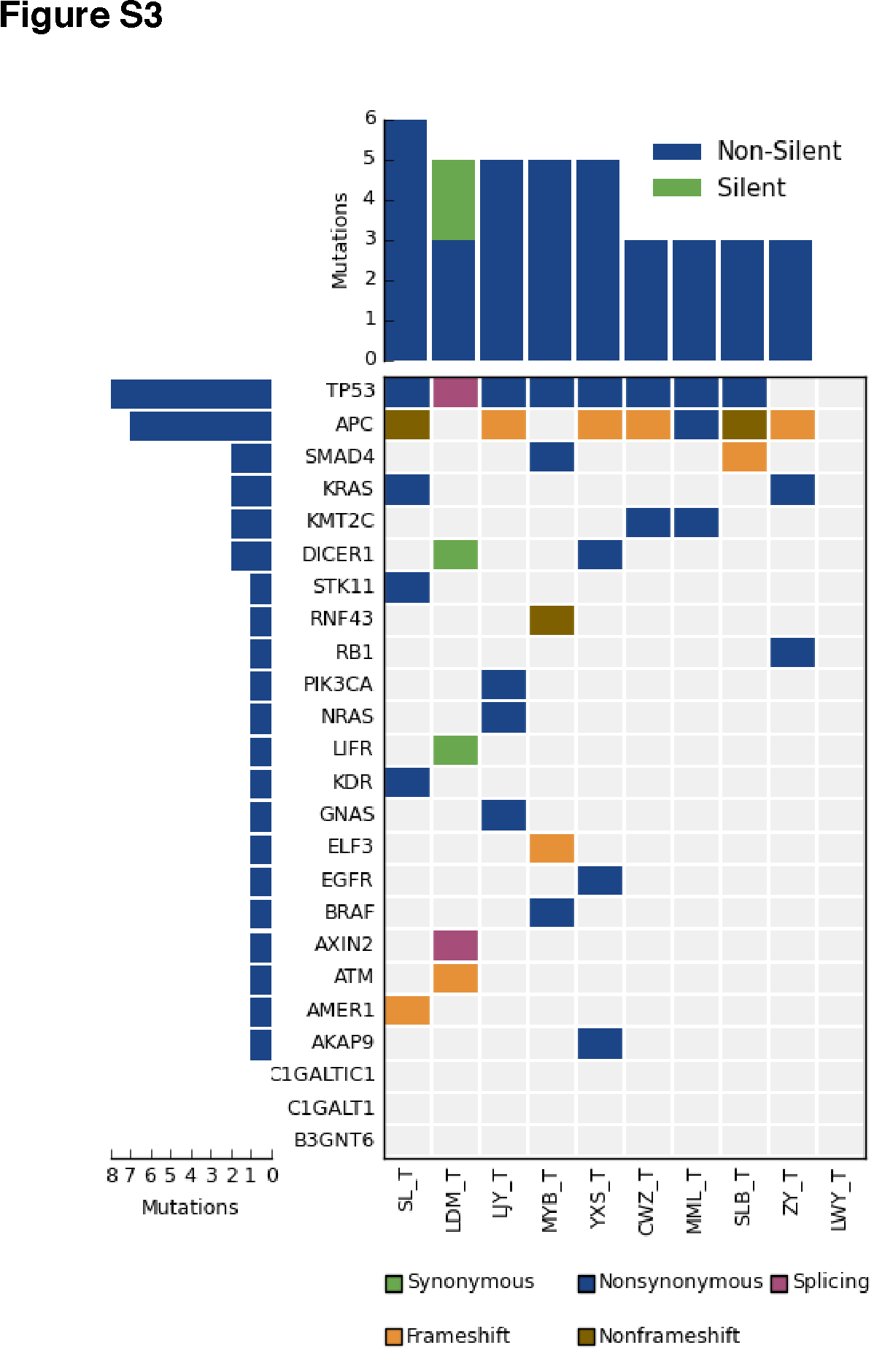

Supplement: Supplementary file 3 [file JCMM-22-4875-s003.tif]

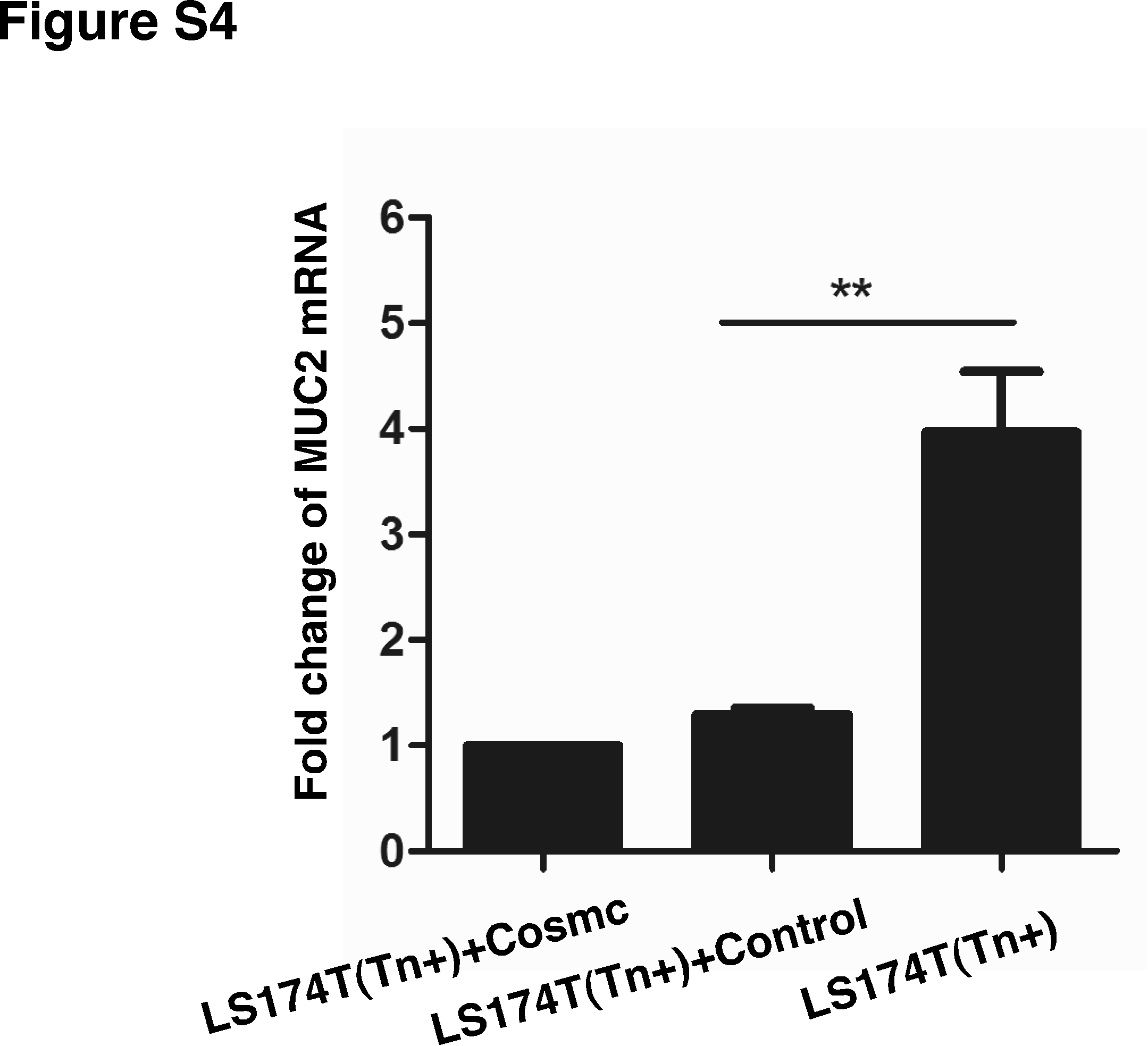

Supplement: Supplementary file 4 [file JCMM-22-4875-s004.tif]
