# Supplementary material for: Aberrant O‐glycosylation contributes to tumorigenesis in human colorectal cancer
Source: J Cell Mol Med. 2018 Jul 12;22(10):4875–85. doi: 10.1111/jcmm.13752 (PMC6156240; doi:10.1111/jcmm.13752)
Supplement: Supplementary file 5 [file JCMM-22-4875-s005.docx]

**Supporting information**

**Methods**

Total RNA was extracted from frozen human CRC tissues and LS174T cells using TRIzol reagent (Invitrogen, CA, USA), according to the manufacturer’s instructions. The RNA quality was assessed by a NanoDrop 2000 spectrophotometer (Wilmington, USA). ppGalNAc-Ts and MUC2 mRNA levels were measured by qPCR using SYBR Premix (Applied Biosystems) on the 7500 Sequence Detection System (Applied Biosystems). GAPDH was used as an internal control.

The sequences of all primers were as follows:

MUC2 F 5'-GAGGGCAGAACCCGAAACC-3'

R 5'-GGCGAAGTTGTAGTCGCAGAG-3'

ppGalNAc-T2: F 5’- ACTACAGCAATGATCCTGAGG-3’

R 5’- TCGATGATGGGTGACACAACC-3’

ppGalNAc-T3: F 5’-ACACTCGACCTCCTGAATGTA-3’

R 5’-ATCATGTAAGTACTCATCTACACTAG-3’

ppGalNAc-T6: F 5’-CCAGCACAGAGGAGCACCTA-3’

R 5’- CGTGGAAGCACTCACAGTGG-3’

ppGalNAc-T12: F 5’- TGACTACAGTGATAGAGAGCACC-3’

R 5’- TCCTCTTCATGGATCCTCTGC-3’

ppGalNAc-T14: F 5’- AGGGTCAAAGAGGACTACACG-3’

R 5’- CCAGCTATGATAGGAGTCCTG-3’

**Supplementary Figure Legends**

**Supplementary Figure 1**.Different pattern of Tn antigen staining in human colorectal cancer samples.

**Supplementary Figure 2**.The changes of a range of ppGalNAc-Ts (ppGalNAc-T2, -T3, -T6, -T12, -T14) mRNA levels in frozen human colorectal cancer tissues (Tn-positive=15, Tn-negative=8). All p>0.05.

**Supplementary Figure 3**. An exome-sequence analysis of Tn-positive (n=10) colorectal cancer tissues found no mutations in T-synthase, Cosmc or C3GnT.

**Supplementary Figure 4**.The changes of MUC2 mRNA levels in LS174T cells transfected with WT Cosmc or blank vector. **p<0.01
